# Mechanical adaptation of trabecular bone morphology in the mammalian mandible

**DOI:** 10.1038/s41598-018-25597-0

**Published:** 2018-05-08

**Authors:** Peter J. Watson, Laura C. Fitton, Carlo Meloro, Michael J. Fagan, Flora Gröning

**Affiliations:** 1 Medical and Biological Engineering Research Group, School of Engineering and Computer Science, University of Hull, Hull, HU6 7RX UK; 2 Centre for Anatomical and Human Sciences, Department of Archaeology and Hull York Medical School, University of York, York, YO10 5DD UK; 3 Research Centre in Evolutionary Anthropology and Palaeoecology, School of Natural Sciences and Psychology, Liverpool John Moores University, Liverpool, L3 3AF UK; 4 Arthritis and Musculoskeletal Medicine Research Programme, School of Medicine, Medical Sciences and Nutrition, University of Aberdeen, Aberdeen, AB25 2ZD UK

## Abstract

Alveolar bone, together with the underlying trabecular bone, fulfils an important role in providing structural support against masticatory forces. Diseases such as osteoporosis or periodontitis cause alveolar bone resorption which weakens this structural support and is a major cause of tooth loss. However, the functional relationship between alveolar bone remodelling within the molar region and masticatory forces is not well understood. This study investigated this relationship by comparing mammalian species with different diets and functional loading (*Felis catus, Cercocebus atys, Homo sapiens, Sus scrofa, Oryctolagus cuniculus, Ovis aries*). We performed histomorphometric analyses of trabecular bone morphology (bone volume fraction, trabecular thickness and trabecular spacing) and quantified the variation of bone and tooth root volumes along the tooth row. A principal component analysis and non-parametric MANOVA showed statistically significant differences in trabecular bone morphology between species with contrasting functional loading, but these differences were not seen in sub-adult specimens. Our results support a strong, but complex link between masticatory function and trabecular bone morphology. Further understanding of a potential functional relationship could aid the diagnosis and treatment of mandibular diseases causing alveolar bone resorption, and guide the design and evaluation of dental implants.

## Introduction

Alveolar bone encloses the tooth roots to provide an attachment site for the periodontal ligament and thus secure anchorage of the teeth. In addition, alveolar bone and the underlying trabecular bone provide structural support against the mechanical loads induced during mastication. Both alveolar and trabecular bone undergo continuous remodelling and optimization in response to these mechanical loads in order to maintain strength and prevent tissue damage^1^. However, this remodelling process can be affected by common diseases such as osteoporosis^2,3^ and periodontitis^4^, which cause a decrease in trabecular bone volume^5^ and a reduction in height of the alveolar ridge^6^. This reduced structural support can lead to instability of the teeth and eventually tooth loss. Tooth loss is often followed by an irreversible process of further alveolar bone resorption that spreads throughout the alveolar ridge^7^, increasing the risk of further tooth loss. The loss of teeth not only produces subsequent difficulties in chewing but has also been reported to have a negative impact on oral health^8–10^.

In an aging population, alveolar bone resorption has the potential to become a major future healthcare problem. For example, Frencken *et al*.^11^ reported that approximately 10% of the global population are affected by severe periodontitis, with dental care accounting for 5–10% of the expenditure in high-income industrialised countries^12^. Current treatments include dental implants and dentures to replace lost teeth, and although chewing ability is improved, they do not reverse the bone resorption process^13^. In addition, dentures are limited in their ability to replace the functionality of natural teeth, which has significant impact on the quality of life of the patient. The use of bisphosphonates has been reported to inhibit the rate of resorption in the case of periodontitis^14–16^, while the reduced alveolar height has been corrected using bone grafts^17–19^. However, in order to establish the cause of alveolar bone resorption and effective treatment strategies, it is essential to understand the influence of masticatory forces upon bone remodelling within the mandible.

Experimental studies have observed a functional relationship between the masticatory forces and alveolar bone remodelling in rats. For example, swopping to a soft-food diet induces a reduction in masticatory forces and has been accompanied by a decrease in alveolar bone volume^20,21^. Conversely, application of a bite block, which exerted a low continuous force along the molar row, has also been attributed to an increase in the thickness of the cortex within the alveolar process^20^. Milne *et al*.^22^ reported that osteopenia occurred with the application of an orthodontic device which caused stress shielding and a reduction in occlusal loading. In addition, the structural characteristics of alveolar bone have also been observed to alter during molar eruption within pigs^23^. Despite the potential use of this functional relationship to reduce alveolar bone resorption through loading of orthodontic devices^24^ or masticatory muscle exercises, this complex interaction is still not fully understood^25^.

The remodelling of mandibular bone has been proposed to follow the “mechanostat” model of bone regulation^26,27^, whereby bone is either formed or resorbed (i.e. remodelled) in response to mechanical strains induced by external forces, and indeed the morphologies of the cortex within the human corpus and symphysis have been linked to strains generated by mastication^28–31^. However, Lad *et al*.^32^ observed evidence of bone remodelling in regions of a cercopithecoid mandible which are known to experience relatively low strains. Thus, previous attempts to understand the link between bone remodelling and mechanical strain are contradictory. In addition, these observations are limited to the external morphology, leaving the link between masticatory forces and underlying trabecular bone morphology within the mandible to be explored.

The trabecular bone in the post-canine region is of particular interest as this is generally where the highest masticatory loads are found. Histomorphometric analyses have characterised the trabecular architecture of the molar region within both the human maxilla^33,34^ and mandible^34–37^. Ontogenetic development of the mandible has also been analysed, providing detailed information regarding the change in architecture and mineralisation of trabecular bone between pre- and neo-natal pigs^38,39^. This re-organisation of trabeculae in the corpus is reported to correspond to the onset of mechanical loading^38^. Liu *et al*.^40^ observed that any disruption of normal occlusal function can lead to changes in trabecular structure in the rat mandible. Although such histomorphometric studies suggest a functional relationship exists between remodelling of the molar trabecular bone and masticatory forces, they are limited to the analysis of a single species. Despite its potential to further our understanding of molar bone remodelling, a comparison of the trabecular architecture between different species has yet to be attempted.

Differences in masticatory loads between species are reflected in the morphology of the teeth and temporomandibular joints (TMJs) in mammals. For instance, carnivores have blade-like molars with TMJs that limit lower jaw motion to the sagittal plane, enabling them to cut through meat^41^. In contrast, herbivores have molars with flat occlusal surfaces and TMJs that permit transverse movements of the lower jaw, enabling them to grind their food^42^. Therefore, the aforementioned functional relationship would suggest that the trabeculae adjacent to molars in carnivores will be preferentially aligned to facilitate vertical load transfers, while they will be optimised to resist shear forces in herbivores. In addition to differences between species, there are functional differences between post-canine teeth within a species. For example, the premolar teeth in the lower jaw of the rabbit have vertically aligned roots, whereas the molar roots have a posterio-lateral orientation^43^. This suggests different occlusal forces in the molar versus the premolar region, and may lead to varying trabecular structures along the post-canine tooth row.

**Figure 1:**
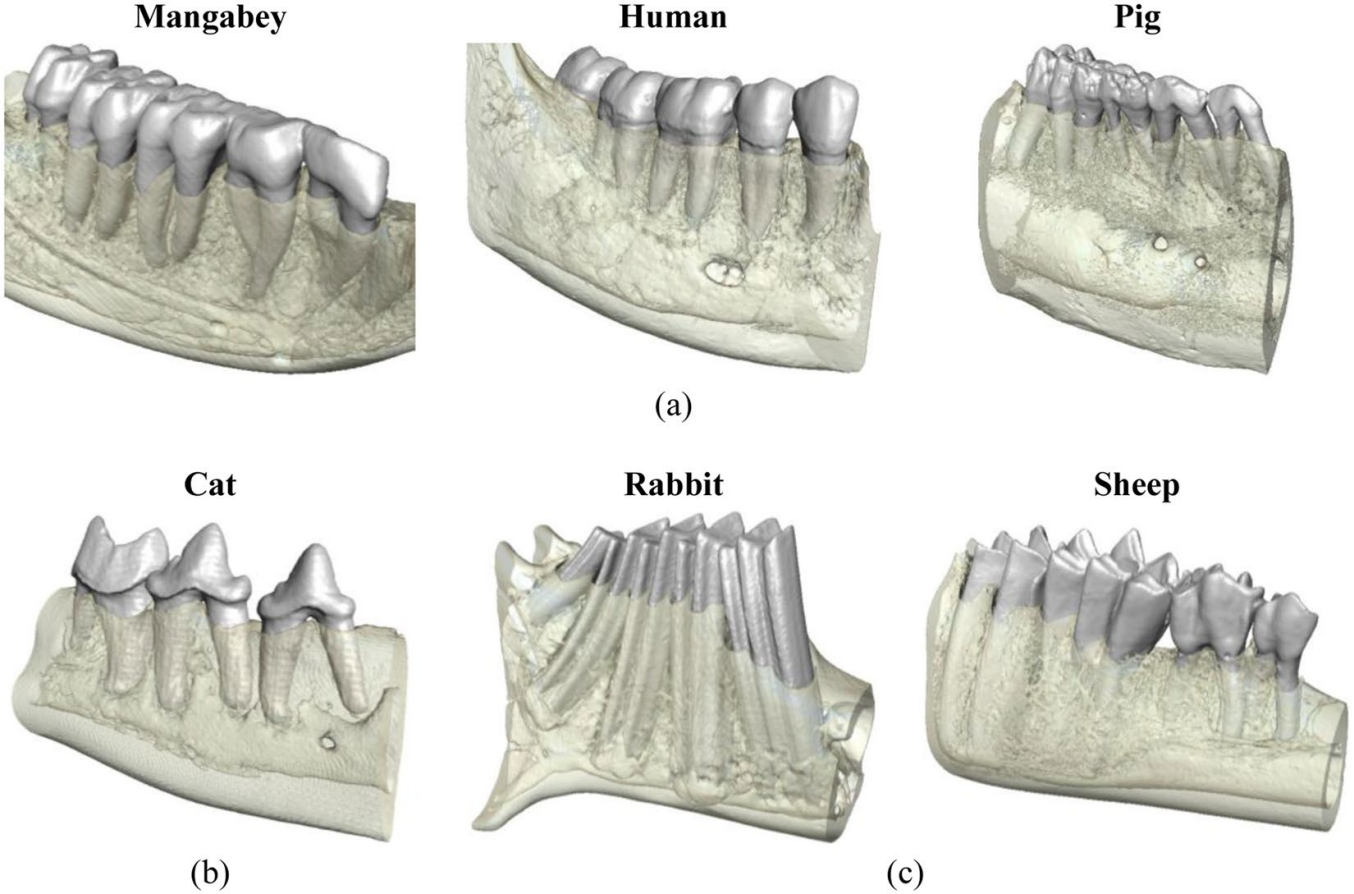
3D visualisations of the post-canine mandibular corpus of the species included in the histomorphometric analysis. These species display diverse molar shapes and functions, namely: (**a**) molars with flattened or rounded cusps – mangabey, human and pig; (**b**) blade-shaped molars – cat; (**c**) flattened molars with ridges – rabbit and sheep.

This study investigated the relationship between masticatory loads and the internal bone architecture around the tooth sockets within the post-canine region in mammalian species with very different diets and molar functions. The species analysed were the cat (*Felis catus*), sooty mangabey (*Cercocebus atys*), human (*Homo sapiens*), domestic pig (*Sus scrofa*), wild European rabbit (*Oryctolagus cuniculus*) and domestic sheep (*Ovis aries*). Molar shape was used as a proxy for functional loading, specifically: blade-shaped molars for shearing in specialised carnivores (cat)^44^; bilophodont molars which, due to wear, possess flattened cusps and ring shaped enamel ridges (mangabey) and simple bunodont molars with low rounded cusps (human and pig), which are suited for crushing and grinding^45–47^; and high crowned selenodont (sheep) and lophodont (rabbit) molars with ridges, primarily for grinding as part of an herbivorous diet^42,44^ (see Fig. 1). Consequently, these species exhibit a variety of feeding behaviours, for example mastication in the cat is dominated by vertical jaw movements (with slight medio-lateral movements) which enables food to be sliced^41,48^. In contrast, pigs process food through bilateral biting, with a more pronounced medial jaw movement in order to pierce, crush and grind food particles^45,49,50^. However, their jaw movements are irregular between consecutive bite cycles, sometimes moving laterally or even without any transverse movement^49^. Masticatory patterns of the human and mangabey are also known to be influenced by material food properties, with the mangabey displaying increased vertical but lower medio-lateral jaw excursions when processing harder foods^51^. The effects on human mastication has been shown to be more variable^52–54^. Rabbit mastication is typical of other herbivores, with a marked medio-lateral jaw movement in order to crush and grind food positioned between opposing transverse ridges of the upper and lower molars^42,55,56^. However, sheep mastication is suggested to be different and has been likened to a cutting process, characterised by compression followed by shearing movements, rather than solely a grinding action^57,58^.

In the first instance we performed two histomorphometric analyses to test the following hypotheses: species with different functional loading on their post-canine teeth will have statistically significant differences in trabecular structures (Hypothesis 1); and, species with similar functional loading on their post-canine teeth will not have statistical significant difference in trabecular structures (Hypothesis 2).

There is evidence to suggest that some species within this selection utilise different molars during the processing of food. For example, the mangabey is reported to bite hard seeds near the P_4_ - M_1_ region^59,60^, suggesting different structural adaptations of the internal bone may exist between this region and that of the M_1_ – M_3_ region. Similarly, the premolar and molar root alignment in the rabbit could suggest differing functional loading in the two regions^43^. Therefore, the study included a further histomorphometric analysis that tested the hypothesis; internal bone volume will vary along the post-canine tooth row in species with different functional loads on the premolar and molar teeth (Hypothesis 3).

Allometry is an important consideration when investigating the variation of histomorphometric traits^61^ and is known to influence the morphometry of the primate and sheep mandible^62,63^. Thus, due to the differing mandibular sizes of the species analysed, allometry was also considered in this study.

## Results

### Analysis of trabecular architecture

**Figure 2:**
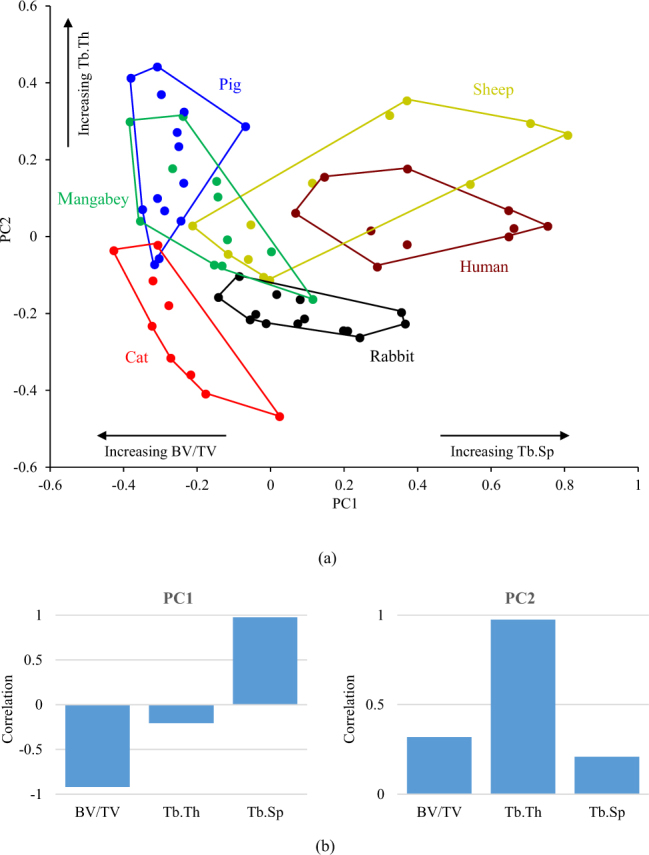
Results of (**a**) the PCA of three trabecular parameters within the RTT bone, and (**b**) the correlation of the three measures to principal components 1 (PC1) and 2 (PC2).

A principal component analysis (PCA) of the bone volume (BV/TV, BV = bone volume, TV = total volume), trabecular thickness (Tb.Th) and trabecular spacing (Tb.Sp) for the bone between the roots of two adjacent teeth (RTT) showed that histomorphometric variance (var.) could be summarised by the first two principal components (PC) vectors (PC1 explained 68.68% var. and PC2 29.93% var.). The scatterplot showed the cat and the rabbit to be considerably distinct from all the other species (Fig. 2(a)), while overlap occurred between the pig and mangabey. The sheep overlapped with both the mangabey and human. PC1 was largely correlated with Tb.Sp (positive correlation) and BV/TV (negative correlation), while PC2 was positively correlated with Tb.Th (Fig. 2(b)).

Non-parametric MANOVA demonstrated that the cat and the rabbit were both significantly different from all the other species in their histomorphometric parameters (Table 1). Mangabey was not significantly different from either the pig or sheep, while there was also no difference between the sheep and human.

**Table 1:** The results of a non-parametric MANOVA to calculate statistical significance between the species within the RTT bone (p < 0.05).

	Cat	Mangabey	Human	Pig	Rabbit	Sheep
Cat	—	**0.008**	**0.002**	**0.002**	**0.002**	**0.002**
Mangabey		—	**0.003**	0.345	**0.002**	0.104
Human			—	**0.002**	**0.003**	0.651
Pig				—	**0.002**	**0.003**
Rabbit					—	**0.005**
Sheep						—

**Figure 3:**
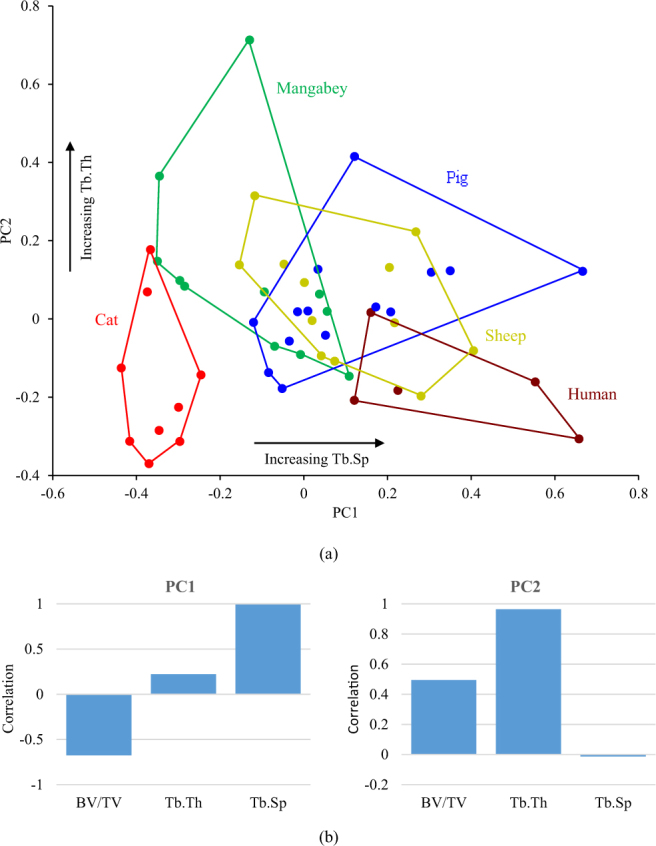
Results of (**a**) the PCA of three trabecular parameters within the RST bone, and (**b**) the correlation of the three measures to principal components 1 (PC1) and 2 (PC2).

A PCA for the trabecular architecture of the bone between the roots of a single tooth (RST) also showed that histomorphometric var. could be summarised by the first two PC vectors (PC1 explained 59.81% var. and PC2 33.27% var.). The scatterplot displayed in Fig. 3(a) showed less separation of the species, when compared to the observations in the RTT bone. The cat was the only species that separated completely from a large cluster, in which the sheep specimens overlapped with all the other species. PC1 had a large positive correlation with Tb.SP, and PC2 was predominately positively correlated with Tb.Th (Fig. 3(b)). BV/TV did not correlate strongly with either component.

A non-parametric MANOVA performed on the RST confirmed the separation of the cat, and to a lesser extent the mangabey, which was significantly different from the pig and human (Table 2). There was no statistical difference between the pig and human, while the sheep was not significantly different from the human, pig or mangabey.

**Table 2:** The results of a non-parametric MANOVA to calculate statistical significance between the species within the RST bone (p < 0.05).

	Cat	Mangabey	Human	Pig	Sheep
Cat	—	**0.003**	**0.007**	**0.001**	**0.002**
Mangabey		—	**0.008**	**0.008**	0.191
Human			—	0.084	0.098
Pig				—	1
Sheep					—

The impact of allometry was investigated via non-parametric correlation (due to non-normality of data) between mandibular length and the trabecular parameters. Within the RTT dataset, mandibular length had no significant impact on PC1 (r_spearman_ = −0.16, p = 0.18) or PC3 (p = 0.32), but it did impact on PC2 (r_spearman_ = 0.73, p < 0.005). Within the RST dataset, mandible length correlated positively with PC1 (r_spearman_ = 0.5911, p < 0.001) and negatively with PC3 (r_spearman_ = −0.47448, p < 0.001) but not with PC2 (p = 0.0069).

### Analysis of internal bone and tooth root volume

**Figure 4:**
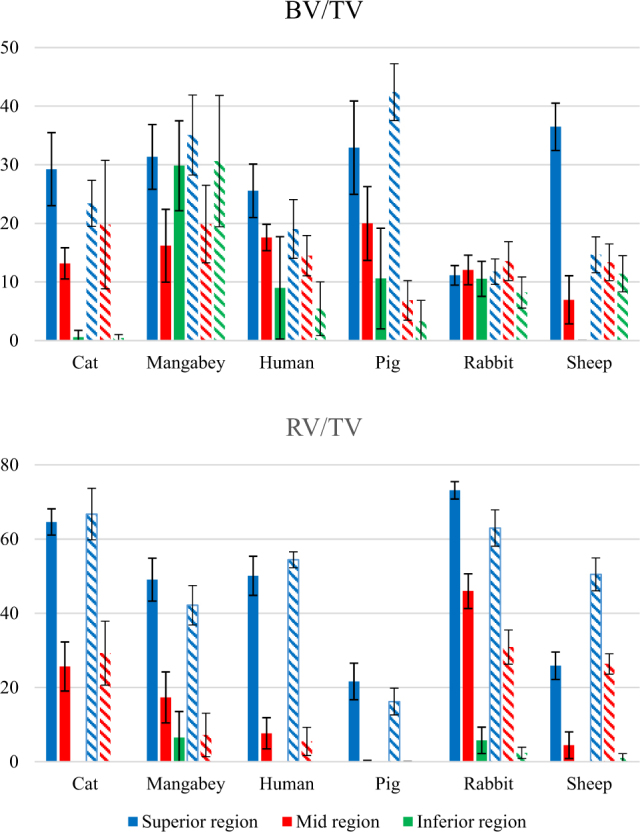
The BV/TV and RV/TV calculated within the superior, middle and inferior regions throughout the premolar (shown as solid bars) and molar (shown as hatched bars) regions. The error bars indicate ±1 standard deviation of the mean value.

Volumes of interest (VOI) were created which captured the maximum volumes of internal bone and tooth root when progressing in an anterio-posterior direction through the molar region, without encroaching the border of the cortex. These VOI tapered and curved to follow the form of the molar region (for further details of the VOI construction see Materials and Methods). Intra-specific analysis of the premolar and molar volumes of interest (VOI) revealed the rabbit to contain relatively consistent BV/TV within the superior, middle and inferior regions (Fig. 4). The magnitudes of BV/TV were also found to be similar between the premolar and molar VOI (typically ~10%). A similar BV/TV was found between the three regions in the molar VOI of the sheep, although the premolar VOI displayed much greater variance. However, a general trend of decreasing BV/TV moving inferiorly through the corpus was observed in all other species, with the exception of the mangabey, where the BV/TV within the inferior region was similar to that of the superior region (~30–35%).

Inter-specific analysis showed that the BV/TV magnitudes for the rabbit and sheep were typically between ~10–20% in all regions (with the exception of the sheep premolar VOI in the superior region) (Fig. 4). Such consistency was not observed in any other species, for example both the pig and human displayed decreasing BV/TV moving inferiorly through the corpus (in both premolar and molar VOI).

Intra-specific analysis of the tooth root volume fraction (RV/TV, RV = tooth root volume, TV = total volume) showed that the magnitudes within the three separate areas were generally similar between the premolar and molar VOI, within all species expect the sheep (Fig. 4). The mangabey and rabbit were the only species to display a RV/TV within the inferior region of the premolar VOI, indicating they have the longest premolar tooth roots (in relation to the height of the mandibular corpus). The rabbit and sheep contain the longest molar tooth roots.

The impact of allometry on this analysis was again investigated via non-parametric correlation between mandibular length and the calculated parameters. Size had no significant influence on BV/TV for the medium and inferior premolar VOI (r_spearman_ = 0.17, p = 0.22 and r_spearman_ = −0.23, p = 0.088, respectively) but it significantly impacted the superior premolar VOI (r_spearman_ = 0.60, p < 0.001). In the case of BV/TV for the molar VOI, there was a significant correlation between size and superior and medium VOI (r_spearman_ = 0.49, p < 0.001 and r_spearman_ = −0.50, p < 0.001, respectively) but not with the inferior VOI (r_spearman_ = −0.16, p = 0.24).

Allometry had a significant influence on the RV/TV in all the premolar and molar VOI (P < 0.01 in all instances), with the greatest effect on the premolar VOI (r_spearman_ = −0.98, −0.93 and −0.64 for superior, medium and inferior respectively) when compared to molar VOI (r_spearman_ = −0.79, −0.47 and −0.29 for superior, medium and inferior respectively).

## Discussion

This is the first study to investigate the relationship between the internal architecture of the mandibular molar region and mastication through a comparison of the trabecular structure of mammalian species with different diets and molar function (e.g. grinding versus crushing or vertical cutting/shearing) (Fig. 1). Due to the complexity of determining the exact functional loading in each of the species analysed, which often requires complex computational modelling^43^, this study used molar shape as a proxy for functional loading.

A PCA of the trabecular bone within the RTT and RST bone demonstrated that the cat, mangabey, human and rabbit (i.e. all species that were represented by specimens with adult dentition) form separate groups from each other within the morphospace (Fig. 2(a) and Fig. 3(a)). Non-parametric MANOVA confirmed that a statistically significant difference in trabecular morphology exists between these species (Tables 1 and 2). This confirms that species with different functional loading contain contrasting trabecular architectures (Hypothesis 1). Although there is separation between the mangabey and human (species that exhibit both crushing and grinding), this could be related to the specialisation of the mangabey in hard object feeding^46^. This will generate larger vertical forces when compared to the human, whose mastication is adapted to process a diverse range of foods.

This finding is consistent with the “mechanostat” theory of bone regulation^26,27^, for example mastication through crushing food items will invoke a predominately vertical force transfer through the tooth root and surrounding alveolar bone. In contrast, mastication through grinding food items will transfer additional horizontally directed forces through the alveolar process. Consequently, as trabeculae are suggested to align to the principal strains associated with mechanical forces^64,65^, differing structures will be created by the two modes of functional loading. This is reflected by the PCA results for the RTT bone, which show that the cat is characterised by a high bone volume comprising of thin, closely spaced trabeculae, whereas the human contains a denser population of thicker, well-spaced trabeculae (Fig. 2(a)).

These observations are supported by some experimental studies of the functional relationship between masticatory forces and molar trabecular bone. For example, Lui *et al*.^40^ reported that structural changes in molar trabeculae were instigated by an appliance which reduced occlusal stimuli, within the rat. However, this has not been observed consistently, with Mavropoulos *et al*.^20^ reporting that no significant adaption of the trabecular structure was found with a bite block that exerted a low continuous force within the same species. Similarly, although trabecular adaptation was observed when inhibiting load transfer through molar extraction within the growing pig, the changes were not found to be significant^23^. However, it has been suggested that trabecular adaption may only be observed when mastication forces are applied in short bursts, followed by a recovery period^20^, and/or after a sufficient time frame^23^, which possibly accounts for these contrasting observations to this study.

Contrasting trabecular structures within the molar region have also been observed between soft and hard food eaters within a single species^20,21,23^. However, despite this link between diet and molar morphology, it is not possible to interpret our results in terms of adaptations to specific diets. Although mangabey’s have a molar morphology capable of crushing their stress resistant food^46^, it is not possible to determine a single soft/hard food diet for all of the species analysed. For example, the rabbit is known to feed on a diet containing a mixture of both soft and hard foods^42^.

Despite separating species with contrasting functional loading, the PCA of the RTT and RST bone failed to group species with similar functional loading (Figs 2(a) and 3(a)). Furthermore, non-parametric MANOVA reported a statistical significant difference between the RTT trabecular structure of the sheep and rabbit (Table 1), although there was no significant difference between the mangabey and pig in the RTT bone, and between the pig and human within the RST bone (Table 2). This suggests rejection of Hypothesis 2, but the hypothesis is dependent on species with similar molar functions producing comparable masticatory loads transfers during mastication. This is not certain to be the case and indeed other factors may influence trabecular adaptation, for instance mandibular torsion has been suggested to influence alveolar bone growth in pigs^23^. Therefore, Hypothesis 2 cannot be rejected with confidence with the current data.

The pig and sheep were the only species to form overlays within the PCA, and often overlapped more than one species; for example the sheep was positioned over the data of the mangabey, human and pig within the RST bone (Fig. 3(a)). This could be related to ontogeny since these specimens were obtained from an agricultural source, and as a result the pig and sheep specimens were of sub-adult age (as reflected by their dentition). The trabecular structure within the corpus has been found to alter during the development of dentition in pigs^23^, therefore the analyses employed here are likely to capture a mixture of partially and fully optimized internal bone.

Analysis of internal bone structures did not show a variation in BV/TV alone the post-canine row in species which are predicted to have differing premolar and molar force transfers, therefore Hypothesis 3 is rejected. Although the literature reports that the mangabey uses the P_4_ - M_1_ region to bite hard seeds^59,60^, comparative regions for the premolar and molar VOI showed similar BV/TV values (Fig. 4). Similarly, despite the root alignment in the rabbit suggesting differing functional premolar and molar loading^43^, once again BV/TV values were consistent along the post-canine row. The pig and sheep were the only species to display different premolar and molar BV/TV magnitudes, although again this may be related to the lack of adult dentition in the analysed specimens.

The mangabey did not display a consistent reduction in BV/TV through the mandible depth, with a relatively high value in the inferior region compared to that of the superior region (Fig. 4). This is a possible indication of a hard food diet, as some species which crush hard foods have a thicker cortex at the base of the mandibular ramus^31,66^, which would be advantageous for resisting the high forces generated during mastication of hard food items. A trabecular network within the inferior region of the mangabey mandibular corpus would provide additional structural support against such loading.

This study has observed a link between the organisation of the molar trabecular and functional loading within the species that were represented by specimens with adult dentition (cat, mangabey, human and rabbit). A clear link could be not be determined for the sub-adult species analysed (pig and sheep), but this possibly highlights the complexity of this relationship. It should be noted that this study used functional loading as a reflection of molar function, rather than solely based on masticatory adaptions (as in many experimental studies). For example, pigs and rabbits have been shown to follow similar mandibular excursions during chewing^42,45,49,55,56^, therefore based on masticatory pattern, it might be presumed that the molar trabecular structures within the two species will be similar. However, as their molar morphology suggests, their diet consists of different food consistencies, and so the resulting difference in occlusal force transfers will produce contrasting trabecular structures (as was observed). As this study is an inter-species histomorphometric analysis it is important to note that the observed relationships could be influenced by phylogenetic signal present in the data. Due to the low sample size it is impossible to employ comparative methods within a robust statistical framework (see Blomberg *et al*.^67^), and only future work based on more interspecific data might clarify such an issue. The results presented support strong differences between species belonging to the same orders in particularly primates for both the RST and RTT bone values (Tables 1 and 2). Artiodactyls (sheep and pig) showed no differences in the RST bone values but strong and significant differences in the RTT.

In conclusion, this paper has observed a link between the internal bone morphology within the molar region and functional loading on the molars. Statistical significance in trabecular architecture was observed between species with contrasting load transfers, with the divergence between species with similar loading possibly being attributed to inclusion of individuals with sub-adult dentition within the analysis. The validity and strength of this link could be explored further through computational modelling to predict the forces generated by differing masticatory patterns, and calculating the corresponding mechanical strains. Developing our understanding of this relationship has a direct clinical application in the investigation of the cause and potential treatment of periodontitis, along with other mandibular diseases. This knowledge can also aid the design and evaluation of dental implants, particularly in terms of implant stability, through furthering our understanding of how mandibular bone remodels to altered masticatory forces post-implantation.

## Materials and Methods

### Analysis of trabecular architecture

All specimens contained adult dentition (with the exception of the pig and sheep), although their exact ages were unknown. Juvenile pig and sheep specimens were agriculturally sourced, and the rabbits were obtained from culling for routine land management. Adult cat specimens were obtained from the Institute of Veterinary Science, University of Liverpool, while young adult human mandibles were obtained from the Scheuer collection (University of Dundee, Scotland). The adult mangabey used in this study is from a collection curated at Hull York Medical School (HYMS) and has been used previously in developmental studies of craniofacial growth^47,68,69^. None of the specimens analysed were sacrificed for the purpose of this study.

The left and right side of each mandible (hereafter referred to as a hemi-mandible) were scanned with an X-Tek HMX 160 µCT scanner (X-Tek Systems Ltd, UK) using spatial resolutions ranging from 16.6 to 96.0 µm to capture the trabecular bone morphology (Table 3). In instances of visible damaged to the molar region on either hemi-mandible, the affected hemi-mandible was omitted from further analysis. The range of resolutions was a result of the different sizes of hemi-mandibles and sizes of the VOI. A sensitivity test was performed to investigate whether this variability affected the histomorphometric analysis of the trabecular architecture. This consisted of scanning one hemi-mandible of each species at the minimum and maximum resolutions shown in Table 3, and creating comparative RTT and RST VOI (further details of the VOI construction are described below). This was performed along the post-canine row (i.e. creating VOI in both the premolar and molar regions). A subsequent histomorphometric analysis concluded that there was no significant difference in the calculated bone parameters between the two scan resolutions. This finding was consistent for all species and confirmed that differences in scan resolutions did not affect the histomorphometric analysis.

**Table 3:** The number of hemi-mandibles analysed per species, and the total number of RTT and RST VOI used in analysis of the trabecular architecture.

	No. hemi-mandibles (No. of individuals)	µCT Scan resolution (µm)	No. of RTT VOI	No. of RST VOI
Cat	7 (5)	19.2–48.6	13	19
Mangabey	12 (6)	57.1–71.1	42	52
Human	10 (5)	74.9–79.5	30	20
Pig	12 (7)	69.5–96.0	36	48
Rabbit	12 (7)	16.6–47.4	48	—
Sheep	12 (6)	45.5–84.8	36	36

The hemi-mandibles were scanned using a range of resolutions, which were subject to a sensitivity test to ensure that they did not affect the histomorphometric analyses of the trabecular bone. Note that no RST VOI was calculated for the rabbit as post-canine rabbit teeth are single-rooted.

**Figure 5:**
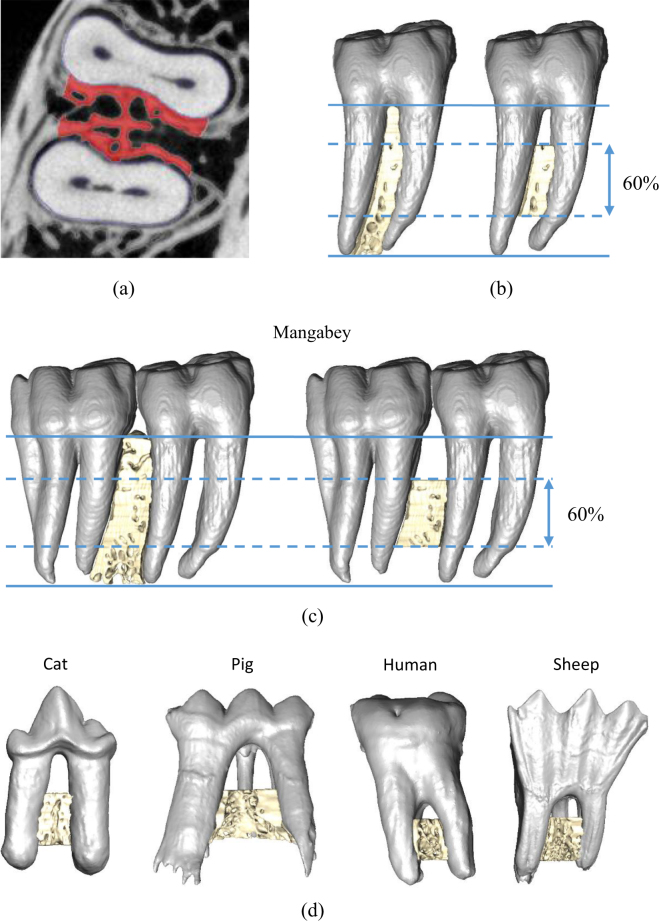
Segmentation of RTT and RST VOIs, showing: (**a**) mapping of the largest area of bone between the medio-lateral borders of the tooth root; (**b**) volumetric representation of the RST bone along the length of the tooth root, and creation of a VOI containing the middle 60% of the bone in the sagittal plane; (**c**) volumetric representation of the RTT bone along the length of the tooth root, and creation of the VOI in the same manner as described in (**b**); and, (**d**) the creation of RTT VOI in other species.

Scan data were imported into the three-dimensional (3D) image processing software AVIZO v6.3 (Visualization Sciences Group, Inc. USA) as a stack of TIFF images. The image stacks were segmented using a ray casting algorithm^70^ which differentiated bone and teeth from non-bone/teeth material, using the grey-level gradient of the image. The teeth and bone were subsequently segmented manually into separate materials, creating a 3D model of each scan, which consisted of the external and internal architecture of the bone, together with the premolar and molar teeth (Figs 1 and 5). Each 3D model was subsequently reoriented so that the occlusal plane was horizontal.

This study analysed the trabecular architecture in the RTT and RST bone. The two types of bone were segmented manually in each hemi-mandible by selecting the bone located within the medio-lateral borders of the respective tooth roots, when viewed in the transverse plane (as illustrated in Fig. 5(a)). By repeating this process for each transverse slice down the length of the tooth root, a series of volumes were created (Fig. 5(b,c)). This process was performed along the post-canine row for each hemi-mandible, except in locations of missing or partially erupted teeth, or where there was visible damage to the tooth crown, tooth root or bone. In order to investigate the trabecular structure, smaller VOI were then defined within the preselected inter-root and intra-root bone volumes by selecting the middle 60% of each (Fig. 5(b,c)). Larger VOI were not used to avoid any artefacts from analysing the bone at the superior and inferior borders. Consequently, species with larger numbers of premolars and molars yielded a greater total number of RTT and RST VOI (Table 3). In addition, only RTT VOI could be created within the rabbit due to their single rooted post-canine teeth.

Each VOI was exported as a stack of TIFF images and imported into ImageJ v1.48, (National Institute of Health, USA)^71^, where a histomorphometric analysis was performed using the plugin BoneJ^72^ to measure the parameters of BV/TV, Tb.Th and Tb.Sp.

Non-parametric Mann-Whitney analyses demonstrated that there were rarely significant differences in the histomorphometric parameters between the left and right VOI for each specimen. Therefore, the data for the left and right hemi-mandibles were combined for each specimen, and then averaged to create a single premolar and molar value for each trabecular parameter. This was performed separately for the RTT and RST datasets. As the analysis also showed few instances of significant difference between premolar and molar VOI for each specimen, both premolar and molar values were included in subsequent analyses.

Both univariate and multivariate statistics were employed to explore the intra- and inter-specific variation of the trabecular parameters: non-parametric Mann-Whitney to test for intra-specific differences; non-parametric MANOVA to test for inter-specific differences; and a PCA to interpret the variation and relationship between the different parameters. All variables were not normally distributed and our non-parametric approach was conservatively supported by permutation tests (via 9,999 permutations). Data analyses were performed in statistical software packages PAST^73^ and R^74^.

### Analysis of internal bone and tooth root volume

A second histomorphometric analysis was performed to investigate the variation in the volume of the internal bone and tooth roots throughout the mandibular body, in particular the variation along the post-canine tooth row and in the superio-inferior direction. The analysis required full dentition in the hemi-mandible, therefore it was only performed on a subset of the original dataset in Table 3, specifically: 4 cat hemi-mandibles (from 4 individuals); 8 mangabey hemi-mandibles (from 4 individuals); 8 human hemi-mandibles (from 4 individuals); 11 pig hemi-mandibles (from 7 individuals); 11 rabbit hemi-mandibles (from 6 individuals); 12 sheep hemi-mandibles (from 6 individuals).

**Figure 6:**
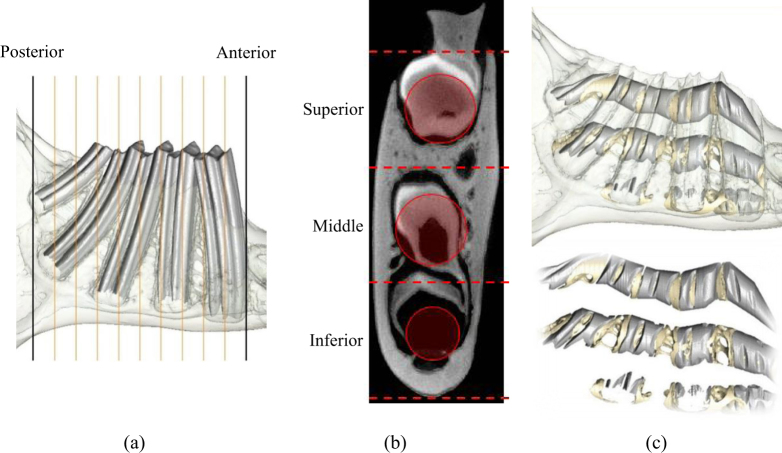
Methodology used to calculate the volume fractions of bone and tooth material throughout the post-canine mandibular body. Construction of extruded volumes through the tooth row, showing: (**a**) division of the tooth row into 10 equally spaced regions using transverse slices between the anterior border of the first premolar, and the inferior borders of the last molar (black lines); (**b**) division of each transverse slice identified in part (**a**) into three equally spaced regions between the superior and inferior borders of the bone. A circular area was defined in the centre of each region, with the largest diameter that did not encroach the medial and lateral cortex; and, (**c**) interpolation between the areas circular areas defined in part (**b**) to create extruded VOI which followed curved trajectories through the superior, middle and inferior regions.

The hemi-mandibles were initially viewed in the sagittal plane and a region created using transverse slices that represented the anterior border of the first premolar, and the posterior border of the last molar (Fig. 6(a)). Additional transverse slices were then identified in between these borders in order to create 10 equally sized sub-regions. Each of these slices were subsequently viewed in the transverse plane and three equal regions (termed superior, middle and inferior region) created between the superior and inferior borders of the mandible (Fig. 6(b)). Within the centre of each region a circular area was defined with the largest diameter that did not encroach the medial and lateral cortex. Consequently, as the size of the mandible varied within each transverse slice, the size of the circular area was different within each region (Fig. 6(b)). An interpolation function within AVIZO was utilised to create an extruded VOI between each of the circular areas in the superior, middle and inferior regions (Fig. 6(c)). Although this successfully excluded the medial and lateral cortex from each VOI, the inter-root bone in the anterio-posterior direction was included. This method enabled construction of VOI which followed curved trajectories in three dimensions, and widened/tapered through thicker/thinner sections of the mandible. This was performed through the whole tooth row in the majority of the species, with the exception of the mangabey and human where, due to the presence of partially erupted 3^rd^ molars, the tooth row was defined between the 1^st^ premolar and 2^nd^ molar.

The VOI were divided into a premolar and molar VOI based on the borders of the premolar and molar roots when viewed in the sagittal plane. The parameters of BV/TV and RV/TV were then calculated in each VOI within AVIZO.
